# Peripheral blood invariant natural killer T cells throughout pregnancy and in preeclamptic women

**DOI:** 10.1016/j.jri.2010.07.003

**Published:** 2010-12

**Authors:** Jennifer Southcombe, Chris Redman, Ian Sargent

**Affiliations:** Nuffield Department of Obstetrics and Gynaecology, University of Oxford, John Radcliffe Hospital, Oxford OX3 9DU, UK

**Keywords:** iNKT cells, Human pregnancy, Preeclampsia

## Abstract

Invariant natural killer T (iNKT) cells are implicated in the pathogenesis of several diseases. They influence both innate and adaptive immune responses through their capacity to rapidly produce large quantities of cytokines upon activation. During pregnancy maternal immunity is biased towards type 2 cytokine production to regulate type 1 cytokines that could be harmful for the developing fetus. This shift to type 2 cytokines does not occur in preeclamptic women and there is an exaggerated maternal inflammatory response which is dangerous for both mother and baby. We have therefore investigated the numbers, phenotype and functional activity of iNKT cells throughout pregnancy and in women diagnosed with preeclampsia. We demonstrate that the numbers of iNKT cells in the peripheral blood do not change between the first, second and third trimesters of pregnancy, but the cells become activated and less able to produce the type 1 cytokine IFNγ. However, iNKT cells are unchanged in preeclamptic women, when compared to normal pregnancy, suggesting that these cells are not primary players in the pathogenesis of the disease.

## 1 Introduction

During pregnancy, the maternal immune system is modified to enable survival of the semi-allogeneic fetus. It was originally proposed that type 2 cytokines (e.g. IL-4, IL-5, IL-6, IL-10) are prevalent, and type 1 cytokines (e.g. IFNγ, IL-2, IL-12, TNFα and TNFβ) are diminished, to prevent the activation of cell mediated immunity and inflammation (Wegmann et al., 1993). However, the finding that pregnancy itself is an inflammatory state has led to a revision of this hypothesis and it is now apparent that both arms of the immune response are intensified, but with a stronger bias towards type 2 than type 1 responses in successful pregnancy (Redman and Sargent, 2003; Chaouat et al., 2007). The causes behind this cytokine shift are not clearly defined, but it is known that the placenta produces type 2 cytokines such as IL-4 and IL-10, as well as progesterone (Sacks et al., 2001; Szekeres-Bartho et al., 2009), which may influence maternal immunity (De Moraes-Pinto et al., 1997; Chaouat et al., 1999).

Preeclampsia is the most common medical complication of pregnancy affecting 3–5% of all pregnancies. The maternal syndrome is characterised by new-onset hypertension and proteinuria which are caused by the excessive release of placenta derived factors into the blood. These factors stimulate an exaggerated maternal systemic inflammatory response of which endothelial dysfunction is a part (Redman and Sargent, 2009). In preeclampsia, the shift away from type 1 responses either fails to occur or is reversed, with increased production of IL-18 and IFNγ relative to normal pregnancy (Germain et al., 2007). This type 1 bias can be seen in T cells (Saito et al., 2007) and more predominantly in NK and NKT-like (CD3+CD56+) cells (Borzychowski et al., 2005). The latter cell type are of particular interest as recently a specific subset of invariant natural killer T cells (iNKT) has been defined, which link innate and adaptive immune responses (Matsuda et al., 2008).

iNKT cells are a unique population that express an invariant T cell receptor (TCR), comprised of Vβ11 and Vα24 chains (Porcelli et al., 1993; Prussin and Foster, 1997). They also share characteristics with NK cells, in that they are cytotoxic through the action of granzymes and perforins, and are potent producers of a wide variety of cytokines (Crowe et al., 2003; Van Kaer, 2004). iNKT cells are important regulators of host defence in microbial and viral infections (Bendelac et al., 2007), and are involved with the development or progression of a range of conditions, such as cancer, autoimmunity or allergy (Godfrey and Kronenberg, 2004).

iNKT cells recognise and are activated by endogenous or exogenous lipid ligands presented by the major histocompatibility complex class I-like molecule, CD1d, on antigen presenting cells (Barral and Brenner, 2007). They can be activated using a pharmacological agent; α-galactosylceramide (α-galcer) which is based on a glycosphingolipid isolated from the marine sponge *Agelas mauritianus*, and has antitumour activity in the mouse B16 melanoma model (Morita et al., 1995; Kawano et al., 1997). Activation with α-galcer leads to the release of both type 1 and type 2 cytokines and the secondary activation of T cells, B cells and NK cells. The capacity of iNKT cells to produce cytokines can be defined by their differential expression of CD4 and CD8. CD4+ iNKT cells have a Th0 cytokine response in that they produce both type 1 and type 2 cytokines, CD8+ iNKT are primarily type 1 cytokine producers and double negative (CD4−/CD8−) iNKT produce type 1 cytokines but only low levels of IL-4 (Exley et al., 1997; Takahashi et al., 2000; Gumperz et al., 2002; Kim et al., 2002; Lee et al., 2002; Takahashi et al., 2002).

Several observations suggest that iNKT cells may play a key role in pregnancy and preeclampsia. First, treatment of pregnant mice with a-galcer results in abortion due to iNKT IFN-γ release (Ito et al., 2000; Boyson et al., 2006). Secondly, iNKT cells play a role in priming Th2 immune responses (Prussin and Foster, 1997; Singh et al., 1999). Thirdly, iNKT cells regulate NK cell activation and proliferation (Carnaud et al., 1999; Eberl and Macdonald, 2000; Metelitsa et al., 2001), cells that are known to be critical for the progression and success of pregnancy.

We have therefore investigated the absolute count, phenotype (CD4, CD8 and CD69 expression) and functional activity (IFNγ production) of iNKT cells in each trimester of pregnancy and in matched trios of non-pregnant, normal pregnant and preeclamptic women. Our aim was to determine whether there are changes in iNKT cells associated with pregnancy, and if so, how they might differ in preeclampsia.

## 2 Methods

### 2.1 Subjects

To study iNKT cells throughout pregnancy, 15 healthy women were recruited in the first trimester, and subsequent samples were taken during the second and third trimesters (Table 1). The mean age of the women was 31.27 (±4.17) years and 40% were nulliparous. Of the women recruited for this study, two developed preeclampsia later in pregnancy but had normal blood pressure at the time of each sample. Data for these two women was included in the study, removing the data did not alter the results. Nineteen preeclamptic women were recruited who presented with ≥90 mmHg diastolic BP on at least two occasions within 24 h and proteinuria ≥500 mg in a 24-h protein urine collection, 50 mg/mmol protein/creatinine ratio or at least 2+ on dipstick testing on two consecutive measurements. Preeclamptic women (15 primiparous and 4 multiparous women) were recruited and matched to 19 non-pregnant women for age (± 4 years) and parity (0, 1–3, 4+), and to 19 normal pregnant women for age, parity and gestational age (±13 days) (Table 2). All cases and controls were not in labour at the time of sampling, and had singleton pregnancies, with no known fetal abnormalities. All control pregnancies progressed normally to term. None of the women had any significant medical history, current or recent illnesses, or were taking medication (excluding contraceptives in the non-pregnant group). These studies were approved by the Oxfordshire Research Ethics Committee C.

### 2.2 Blood collection

Blood samples (20–30 ml) were collected into sodium heparin anti-coagulant (10 U/ml) and peripheral blood mononuclear cells (PBMC) were prepared by density gradient centrifugation over lymphoprep (Axis Shield Diagnostics, Cambridgeshire, UK). Cells were washed twice with PBS/1% FCS and consecutive centrifugation of 800 g and 200 × *g* × 10 min, then used immediately for flow cytometry while the remainder were re-suspended in 10% DMSO in FCS and cryopreserved in liquid nitrogen.

### 2.3 Flow cytometry

The concentrations of T cells and PBMC were determined by flow cytometry using Cytognos absolute count beads (Caltag-MedSystems, Buckingham, UK). Whole blood (50 μl) was incubated with CD3-PeCy5 antibodies (eBiosciences, San Diego, USA) for 15 min then the red blood cells were lysed with FACS Lysing Solution (Becton Dickinson, Oxford, UK) for 15 min. Absolute count beads (50 μl) were added and the number of PBMC or CD3+ve T cells per ml blood was calculated relative to the beads. iNKT cells were identified using six-colour flow cytometry. Antibodies used were FITC anti-human TCR Vβ11 and PE anti-human TCR Vα24 (Beckman Coulter, High Wycombe, UK), PeCy5-anti-human CD3 (Biolegend, San Diego, USA), PeCy7 anti-human CD8α (eBioscience, San Diego, USA), and APC anti-human CD69 and APC anti-human CD4 (Becton Dickinson, Oxford, UK).

PBMC (2 × 10^6^) were incubated with the six antibodies for 20 min at 4 °C in the dark, then washed twice with 1 ml PBS/1% FCS and brief centrifugation at 10,000 × *g* × 5 s. Cells were resuspended in 1 ml PBS/1% FCS and at least 1 million events were acquired immediately on the flow cytometer (Becton Dickinson LSR II). Compensation was determined using FACS compensation beads (Becton Dickinson, Oxford, UK) and gates drawn using Fluorescence Minus One gating (Roederer, 2002). iNKT cells within the PBMC gate were identified as CD3+Vα24+Vβ11+ events. The percentage of iNKT cells within the CD3+ve gate was calculated and related to the known absolute count of CD3+ve cells per ml of blood to determine the iNKT cell absolute count. Data was analysed using FACSDIVA software (Becton Dickinson, Oxford, UK).

### 2.4 Measurement of iNKT cell IFNγ production by ELISPOT

IFNγ production by iNKT cells was measured using an ELISPOT assay (Mabtech, Sweden). PBMC were thawed and cultured for 24 h in human serum media (RPMI 1640 supplemented with 10% human serum (Sera Laboratories International, West Sussex, UK), 1% penicillin-streptomycin (50 IU/ml and 50 μg/ml), 1% glutamine, MEM NEAA, sodium pyruvate and 50 nM 2-mercaptoethanol (Gibco Invitrogen, Paisley, UK)), supplemented with 500 U/ml IL-2 (Chiron, California, USA). Freezing did not affect the number of responsive iNKT cells (data not shown). Live cells were then counted using trypan blue staining and 5 × 10^5^ PBMC added per well to the ELISPOT plates in the presence or absence of 100 ng/ml α-galcer (Proimmune, Oxford, UK). 10^4^ cells per well were incubated with 1:100 dilution of phytohemagglutinin (Sigma, St. Louis, USA) as a positive control. Cells were incubated overnight and IFNγ producing cells were detected as described in the manufacturer's instructions. Spots were counted using an AID ELISPOT plate reader (Autoimmun Diagnostika GmbH, Strassberg, Germany), and spots were analysed using AID Software. Numbers of iNKT cells per 5 × 10^5^ PBMC were determined from the flow cytometry analysis, and therefore percentages of responding iNKT cells were calculated. Each experiment was performed in triplicate (*n* = 3).

### 2.5 Statistical analysis

For the longitudinal study of CD69 expression levels on iNKT cells, Gaussian distribution was confirmed by a Shapiro–Wilk test, and significance values calculated by ANOVA with a Newman–Kewls post test, ‘*’ is *p* < 0.01. Data for iNKT IFNγ production was non-parametric and therefore a Wilcoxon test was performed for statistical analysis, *p < 0.05 and **p < 0.005. Significant differences between maximum blood pressure and birthweight between the preeclamptic and normal pregnant women were detected using a Wilcoxon matched pairs test (ns – non-significant).

## 3 Results

### 3.1 iNKT cell numbers and phenotype throughout pregnancy

**Fig. 1:**
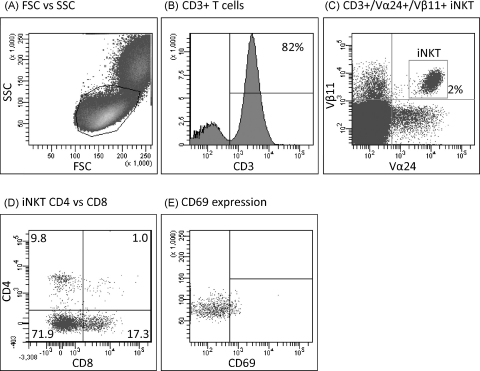
Representative flow cytometry analysis. PBMC were isolated by lymphoprep density preparation then stained with antibodies for flow cytometry. Live PBMC were identified by FSC vs. SSC (A) and iNKT cells were CD3 positive (B) and Vα24 and Vβ11 positive (C). Expression of CD4 and CD8 (D) and CD69 expression (E) was detected. For simplicity, the figure shows CD69 expression on the whole iNKT cell population however in the study CD69 expression was determined on each of the CD4+CD8−, CD4−/CD8+ and double negative populations.

Six colour flow cytometry was used to study iNKT cells in the peripheral blood of normal pregnant women during each trimester of pregnancy. A representative example of the data acquired can be seen in Fig. 1. Lymphocytes were gated on forward and side scatter (Fig. 1(A)), CD3+ve T cells were gated (Fig. 1(B)) and iNKT cells were identified as CD3+Vα24+Vβ11+ cells within the lymphocyte population (Fig. 1(C)). iNKT expression of CD4 and CD8 (Fig. 1(D)) and CD69, an early T cell activation marker (Fig. 1(E)), was also analysed.

**Fig. 2:**
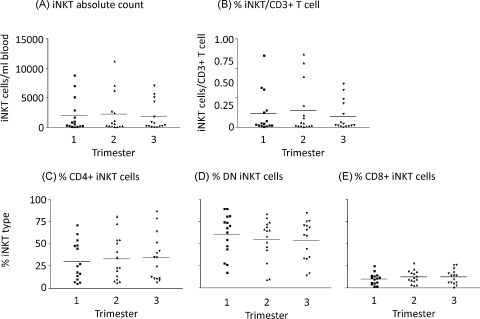
Enumeration and phenotyping of iNKT throughout pregnancy. Flow cytometry determination of the numbers and phenotype of iNKT cells in the peripheral blood of pregnant women, in the first, second and third trimesters of pregnancy. iNKT cells were classified as CD3+Vα24+Vβ11+ cells. Absolute counts of iNKT cells (A) were determined by calculating the number of CD3+ T cells in a whole blood sample, then the proportion of iNKT cells per CD3+ T cells on PBMC prepared by density centrifugation (B) was calculated and this value related to the whole blood T cell count. Antibodies towards CD4 or CD8 identified CD4+ (C), CD4−CD8− DN (D) or CD8+ (E) iNKT cells. Bars represent mean values.

**Fig. 3:**
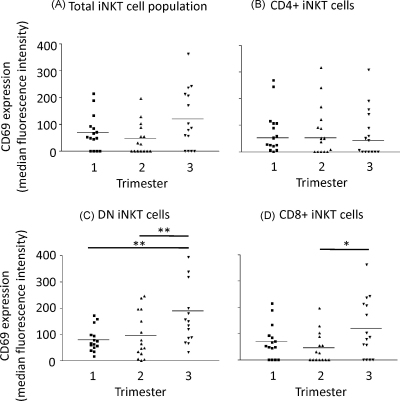
Expression of the activation marker CD69 by iNKT cell populations. Flow cytometry was used to calculate the expression of CD69 (median fluorescence intensity) on the total iNKT cell population (A), CD4+ iNKT (B), DN iNKT (C) or CD8+ iNKT (D). Bars represent median values.

The absolute count of iNKT cells per ml of blood remained constant throughout pregnancy (Fig. 2(A)) as did the relative numbers of iNKT cells measured as a proportion of the T cells (Fig. 2(B)). Percentages of CD4+ve, CD8+ve and CD4−ve/CD8−ve (double negative – DN) iNKT did not alter significantly throughout pregnancy (Fig. 2(C)–(E)). CD69 expression (measured as the increase of median fluorescence intensity) on the total iNKT cell population was slightly increased in the third trimester but this was not statistically significant (Fig. 3(A)). However, when the subgroups of cells were analysed, CD69 expression was significantly increased on the CD4−ve/CD8−ve and CD8+ iNKT cells during the third trimester of pregnancy (Fig. 3(C) and (D)). No changes in this marker were seen on the CD4+ iNKT population (Fig. 3(B)).

### 3.2 IFNγ production by iNKT cells throughout pregnancy

**Fig. 4:**
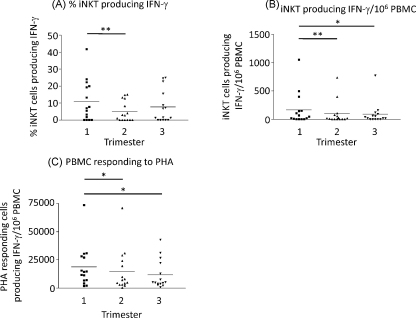
IFNγ production by iNKT cells in response to α-galcer. PBMC samples were treated with 100 ng/ml α-galcer and IFNγ released detected by ELISPOT assay. Known numbers of PBMC were added per well and the number of iNKT cells responding per 10^6^ PBMC calculated (A). Flow cytometry identified the number of iNKT cell per 10^6^ PBMC therefore the % of iNKT cells able to respond to α-galcer was calculated (B). PBMC responses to PHA was determined as a positive control (C). The data is non-parametric and therefore a Wilcoxon test was performed for statistical analysis, *p < 0.05 and **p < 0.005.

The percentage of iNKT cells producing IFNγ during the second trimester of pregnancy was significantly lower than in the first trimester (Fig. 4(A)). The number of iNKT cells per ml of blood producing IFNγ was also significantly decreased during the second and third trimesters compared to the first (Fig. 4(B)). A similar significant decrease in IFNγ production was seen in PBMC stimulated with PHA, as a positive control (Fig. 4(C)).

### 3.3 iNKT cell numbers and phenotype in preeclampsia

**Fig. 5:**
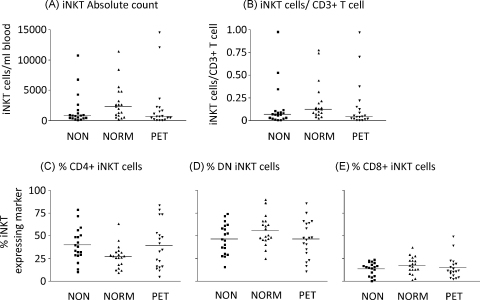
Enumeration and phenotype of iNKT cells in non-pregnant, normal pregnant and preeclamptic women. The absolute count (A) or proportion of iNKT cells in the T cell population (B) was determined by flow cytometry. Antibodies towards CD4 or CD8 identified CD4+ (C), CD4−CD8− DN (D) or CD8+ (E) iNKT cells. Bars represent mean values.

**Fig. 6:**
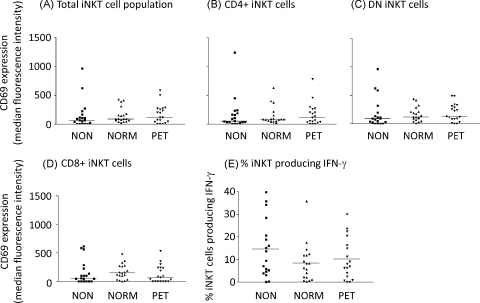
Activation of iNKT cells in non-pregnant, normal pregnant and preeclamptic women. Flow cytometry was used to calculate the expression of CD69 (median fluorescence intensity) on the total iNKT cell population (A), CD4+ NKT (B), DN iNKT (C) or CD8+ iNKT (D). IFN-γ production by iNKT cells in response to α-galcer was determined by ELISPOT as previously described, % iNKT producing IFN-γ (E).

The numbers and phenotype of iNKT cells were measured in preeclamptic women compared to matched normal pregnant and non-pregnant controls. There was no significant difference in the absolute count of iNKT cells per ml of blood or the percentages of iNKT cells in the T cell population between the three groups of women (Fig. 5(A) and (B)). Similarly, there was no change in the proportion of iNKT cells expressing CD4 or CD8 between the three groups (Fig. 5(C)–(E)). iNKT cell expression of the early activation marker CD69 was unchanged between the three groups as a whole (Fig. 6(A)) or within each CD4/DN/CD8 subset (Fig. 6(B)–(D)).

### 3.4 IFNγ production by iNKT cells in preeclampsia

There was a slight reduction in the numbers of iNKT cells producing IFNγ in normal pregnant women compared to women with preeclampsia and the non-pregnant controls but this was not statistically significant (Fig. 6(E)).

## 4 Discussion

Changes in iNKT cell numbers, phenotype and function are associated with progression of various diseases, for example in allergies such as asthma (Matangkasombut et al., 2009), autoimmunities such as systemic lupus erythematosus (Gabriel et al., 2009) and cancer (Molling et al., 2008), often with associated cytokine imbalance. The ability of iNKT cells to drive and modulate immune responses makes them attractive candidates for being key regulators of immunity during pregnancy. Boyson et al. have previously shown that iNKT cells are present in first trimester (7–10 weeks) decidua at a frequency 10 times greater than seen in peripheral blood (Boyson et al., 2002). It is possible that they interact with CD1d expressed on the invasive extravillous cytotrophoblast. Interestingly CD4+ve decidual iNKT cell clones stimulated with CD1d transfected C1R cells (a MHC class I negative EBV transformed B cell line) pulsed with α-galcer showed a strong bias to IFNγ (type 1) production compared to peripheral blood iNKT clones which were biased to IL-4 production (type 2).

In this study we focussed on iNKT cells in peripheral blood as our aim is to study their role in the maternal syndrome of preeclampsia which is a maternal systemic inflammatory disorder of the second half of pregnancy. We first investigated iNKT cells during each trimester of pregnancy, and found that while the number and type of iNKT cells do not change, the cells become activated, as demonstrated by increased CD69 expression on CD4−ve/CD8−ve and CD8+ iNKT cells during the third trimester. IFNγ production is reduced, which is consistent with previous observations showing a shift to a type 2 phenotype on NK and NKT-like cells (Borzychowski et al., 2005) and a type 2 bias in cytokine production in pregnancy (Germain et al., 2007). This data suggests that iNKT cells contribute to this cytokine bias. We were unable to measure the concomitant production of IL-4 due to ethical limitations on the volume of blood we were able to take for these studies, but from the results of Boyson et al. it might be expected that an increase in IL-4 production by iNKT cells would also be seen later in pregnancy. The increased activation of CD4 negative cells, typically type 1 cytokine producers, is paradoxical, and may represent a portion of cells that have been activated but have become anergic in response to chronic stimulation in vivo, a feature of activated human and mouse iNKT cells after long term exposure to α-galcer (Sullivan and Kronenberg, 2005).

From the above results it might be expected that iNKT cells could play an important role in preeclampsia where increased IFNγ production by PBMC is seen compared to normal pregnancy (Germain et al., 2007). However, no differences in iNKT cell number or phenotype were seen between women with preeclampsia and their matched normal pregnant and non-pregnant controls. A slight decrease in iNKT cell production of IFNγ was seen in normal pregnant compared to preeclamptic women, but this was not statistically significant. There has been one previous study of peripheral blood iNKT cells in preeclampsia (Miko et al., 2008). In contrast to our study, it was found that the percentage of activated (CD69+ve) iNKT cells was significantly higher in preeclampsia compared to the matched normal pregnant and non-pregnant controls as was the percentage of iNKT cells producing IFNγ (as determined by intracellular cytokine staining). They did not investigate iNKT cells at other stages of gestation. The differences in results between the studies may be due to differences in the methodology. Miko et al. stained 50 μl whole blood for the iNKT TCR using the 6B11 antibody (Montoya et al., 2007), and analysed at least 1000 iNKT cells, i.e. greater than 20,000 cells/ml, which is very high for iNKT cells. Our data are more consistent with other reports, for example Molling et al. (2005) who show an average of approximately 1000 iNKT/ml blood at 30 years age; our patients had a range of just 46 to 14,563 iNKT cells/ml blood. Miko et al. gated their lymphocytes on forward and side scatter only rather than using CD3 staining as in our study, which could have lead to the inclusion of false positive events in their analysis.

In summary, we show evidence for activation of iNKT cells and a decrease in IFNγ production as pregnancy progresses. However, no significant changes in CD69 expression or IFNγ production were seen between preeclamptic and normal pregnant women, suggesting that iNKT cells are not a major player in the pathogenesis of this condition.

## Figures and Tables

**Table 1:** Patient data for the longitudinal study. Data are shown as mean (± standard deviation).

	1st trimester	2nd trimester	3rd trimester
Gestation (weeks)	12.35 (1.00)	21.70 (1.85)	35.70 (1.24)
Booking bp, mmHg	111/68 (15.56/8.18)	–	–
Birthweight	–	–	3585.80 (478.95)

**Table 2:** Patient details for matched groups of non-pregnant, normal pregnant and preeclamptic women (*n* = 19). Data are shown as mean (± standard deviation).

	Non-pregnant	Normal pregnant	Preeclamptic
Age (years)	30.63 (5.55)	29.42 (4.29)	30.05 (5.02) ns
Nulliparous	79%	79%	79% ns
Gestation (weeks)	–	35.17 (3.59)	35.82 (3.25) ns
Booking bp, mmHg	–	110/65 (10.28/6.96)	115/68 (11.31/10.27) ns
Max bp, mmHg	–	133/78 (13.05/11.01)	164/104 (14.22/7.90) *p* < 0.0005
Max proteinuria, mg/24 h	–	–	2911.36 (3131.62) Range 525–12,801
Birthweight	–	3437.71 (453.42)	2672.32 (868.43) *p* < 0.05

## References

[bib0005] Barral D.C., Brenner M.B. (2007). CD1 antigen presentation: how it works. Nat. Rev. Immunol..

[bib0010] Bendelac A. (2007). The biology of NKT cells. Annu. Rev. Immunol..

[bib0015] Borzychowski A.M. (2005). Changes in systemic type 1 and type 2 immunity in normal pregnancy and preeclampsia may be mediated by natural killer cells. Eur. J. Immunol..

[bib0020] Boyson J.E. (2002). CD1d and invariant NKT cells at the human maternal–fetal interface. Proc. Natl. Acad. Sci. U.S.A..

[bib0025] Boyson J.E. (2006). Gestation stage-dependent mechanisms of invariant natural killer T cell-mediated pregnancy loss. Proc. Natl. Acad. Sci. U.S.A..

[bib0030] Carnaud C. (1999). Cutting edge: cross-talk between cells of the innate immune system: NKT cells rapidly activate NK cells. J. Immunol..

[bib0035] Chaouat G. (1999). Localization of the Th2 cytokines IL-3, IL-4, IL-10 at the fetomaternal interface during human and murine pregnancy and lack of requirement for fas/fas ligand interaction for a successful allogeneic pregnancy. Am. J. Reprod. Immunol..

[bib0205] Chaouat G. (2007). The Th1/Th2 paradigm: still important in pregnancy?. Semin. Immunopathol..

[bib0040] Crowe N.Y. (2003). Glycolipid antigen drives rapid expansion and sustained cytokine production by NK T cells. J. Immunol..

[bib0045] De Moraes-Pinto M.I. (1997). Localization of IL-4 and IL-4 receptors in the human term placenta, decidua and amniochorionic membranes. Immunology.

[bib0050] Eberl G., Macdonald H.R. (2000). Selective induction of NK cell proliferation and cytotoxicity by activated NKT cells. Eur. J. Immunol..

[bib0055] Exley M. (1997). Requirements for CD1d recognition by human invariant Valpha24+ CD4−CD8− T cells. J. Exp. Med..

[bib0060] Gabriel L. (2009). The role of iNKT cells in the immunopathology of systemic lupus erythematosus. Ann. NY Acad. Sci..

[bib0065] Germain S.J. (2007). Systemic inflammatory priming in normal pregnancy and preeclampsia: the role of circulating syncytiotrophoblast microparticles. J. Immunol..

[bib0070] Godfrey D.I., Kronenberg M. (2004). Going both ways: immune regulation via CD1d-dependent NKT cells. J. Clin. Invest..

[bib0075] Gumperz J.E. (2002). Functionally distinct subsets of CD1d-restricted natural killer T cells revealed by CD1d tetramer staining. J. Exp. Med..

[bib0080] Ito K. (2000). Involvement of decidual Valpha14 NKT cells in abortion. Proc. Natl. Acad. Sci. U.S.A..

[bib0085] Kawano T. (1997). CD1d-restricted and TCR-mediated activation of Valpha14 NKT cells by glycosylceramides. Science.

[bib0090] Kim C.H. (2002). Trafficking machinery of NKT cells: shared and differential chemokine receptor expression among V alpha 24(+) V beta 11(+) NKT cell subsets with distinct cytokine-producing capacity. Blood.

[bib0095] Lee P.T. (2002). Distinct functional lineages of human V(alpha)24 natural killer T cells. J. Exp. Med..

[bib0100] Matangkasombut P. (2009). Natural killer T cells and the regulation of asthma. Mucosal Immunol..

[bib0105] Matsuda J.L. (2008). CD1d-restricted iNKT cells, the ‘Swiss-army knife’ of the immune system. Curr. Opin. Immunol..

[bib0110] Metelitsa L.S. (2001). Human NKT cells mediate antitumor cytotoxicity directly by recognizing target cell CD1d with bound ligand or indirectly by producing IL-2 to activate NK cells. J. Immunol..

[bib0115] Miko E. (2008). The role of invariant NKT cells in preeclampsia. Am. J. Reprod. Immunol..

[bib0210] Molling J.W. (2005). Peripheral blood IFN-gamma-secreting Valpha24+Vbeta11+ NKT cell numbers are decreased in cancer patients independent of tumor type or tumor load. Int. J. Cancer.

[bib0120] Molling J.W. (2008). Invariant natural killer T cells and immunotherapy of cancer. Clin. Immunol..

[bib0125] Montoya C.J. (2007). Characterization of human invariant natural killer T subsets in health and disease using a novel invariant natural killer T cell-clonotypic monoclonal antibody, 6b11. Immunology.

[bib0130] Morita M. (1995). Structure–activity relationship of alpha-galactosylceramides against b16-bearing mice. J. Med. Chem..

[bib0135] Porcelli S. (1993). Analysis of T cell antigen receptor (TCR) expression by human peripheral blood CD4−8− alpha/beta T cells demonstrates preferential use of several V beta genes and an invariant TCR alpha chain. J. Exp. Med..

[bib0140] Prussin C., Foster B. (1997). TCR V alpha 24 and V beta 11 coexpression defines a human NK1 T cell analog containing a unique Th0 subpopulation. J. Immunol..

[bib0145] Redman C.W., Sargent I.L. (2003). preeclampsia, the placenta and the maternal systemic inflammatory response—a review. Placenta.

[bib0150] Redman C.W., Sargent I.L. (2009). Placental stress and preeclampsia: a revised view. Placenta.

[bib0155] Roederer, M., 2002. Compensation in flow cytometry. Current Protocols in Cytometry/Editorial Board, J. Paul Robinson, Managing Editor (Chapter 1, Unit 1 14). 10.1002/0471142956.cy0114s22 18770762

[bib0160] Sacks G.P. (2001). Flow cytometric measurement of intracellular Th1 and Th2 cytokine production by human villous and extravillous cytotrophoblast. Placenta.

[bib0165] Saito S. (2007). The role of the immune system in preeclampsia. Mol. Aspects Med..

[bib0170] Singh N. (1999). Cutting edge: activation of NK T cells by CD1d and alpha-galactosylceramide directs conventional T cells to the acquisition of a Th2 phenotype. J. Immunol..

[bib0175] Sullivan B.A., Kronenberg M. (2005). Activation or anergy: NKT cells are stunned by alpha-galactosylceramide. J. Clin. Invest..

[bib0180] Szekeres-Bartho J. (2009). Progesterone in pregnancy; receptor-ligand interaction and signaling pathways. J. Reprod. Immunol..

[bib0185] Takahashi T. (2002). Cutting edge: analysis of human V alpha 24+ CD8+ NK T cells activated by alpha-galactosylceramide-pulsed monocyte-derived dendritic cells. J. Immunol..

[bib0190] Takahashi T. (2000). Analysis of human V alpha 24+ CD4+ NKT cells activated by alpha-glycosylceramide-pulsed monocyte-derived dendritic cells. J. Immunol..

[bib0195] Van Kaer L. (2004). Regulation of immune responses by CD1d-restricted natural killer T cells. Immunol. Res..

[bib0200] Wegmann T.G. (1993). Bidirectional cytokine interactions in the maternal-fetal relationship: is successful pregnancy a Th2 phenomenon?. Immunol. Today.

